# Effect of silver ion-doped-(ZnFe_1.95_Ce_0.05_O_4_/10% graphene) nanocomposite on structural, coercivity, antimicrobial, antioxidant, and anticancer activities

**DOI:** 10.1039/d6ra01681b

**Published:** 2026-05-11

**Authors:** Amina Toumi, Elbadawy A. Kamoun, Heba Y. Zahran, V. Ganesh, Mohammad Abohassan, H. H. Hegazy, Amr Negm, Ibrahim S. Yahia

**Affiliations:** a Department of Health and Laboratory Sciences, College of Medical and Health Sciences, Liwa University P. O. Box 41009 Abu Dhabi United Arab Emirates; b Department of Chemistry, College of Science, King Faisal University Al-Ahsa 31982 Saudi Arabia ekamoun@kfu.edu.sa badawykamoun@yahoo.com +201283320302; c Laboratory of Nano-Smart Materials for Science and Technology (LNSMST), Department of Physics, Faculty of Science, King Khalid University P. O. Box 9004 Abha Saudi Arabia dr_isyahia@yahoo.com +201283320302; d Department of Clinical Laboratory Sciences, College of Applied Medical Sciences, King Khalid University Abha Saudi Arabia

## Abstract

Ag_*x*_-doped Zn_1−*x*_Fe_1.95_Ce_0.05_O_4_/10% graphene (*x* = 0, 0.025, 0.05, 0.075, and 0.1) ferrite nanostructures were prepared using the sol–gel/auto-combustion method. The crystallinity and structure of the samples were analysed using XRD, EDX, FT-IR spectroscopy, SEM, HR-TEM and SAED analyses. The results reveal that the crystallite size and grain size of the prepared samples ranged from ∼35.81 to 59.7 nm. VSMs are measured over an applied magnetic field (*H*) range of ±20 kG. *M*_s_ values seem to be low, while the coercivity values ranged from ∼148.80 to 86.12 Oe. Meanwhile, the biological activity improved with increasing silver content in NPs, affecting component antibacterial activity, scavenging effects, and cytotoxicity in the *Hep-G* cell line. The antimicrobial inhibition of the nanocomposite was recorded at 71% against *Gram-positive* bacteria and 5% against *Gram-negative* bacteria, while no activity was recorded against fungi. Notably, the antioxidant effect was promising, as the tested NC exhibited DPPH scavenging activity. Increasing the silver content in NCs by adding 0.05 Ag decreased the IC_50_ by 41.5%, and doubling the Ag content further reduced it by 79.5%. Overall, silver had a significant impact on cancer cell viability since the IC_50_ decreased by 71% with 0.05 Ag added to the NCs and by 80% with 0.1 Ag added. The prepared NCs exhibit antibacterial, antioxidant, and anticancer properties, making them suitable biomaterials for versatile medical applications.

## Abstract 2

Preparation route of Ag_*x*_Zn_1−*x*_Fe_1.95_Ce_0.05_O_4_/10% graphene nanostructured using sol–gel/auto-combustion method.

## Graphical abstract

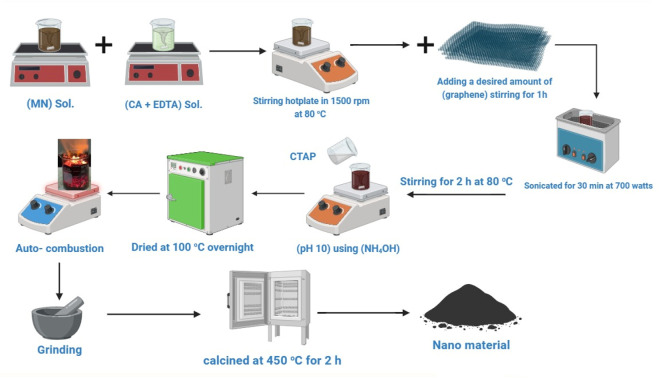

## 1 Introduction

Iron oxides and other metallic components form spinel nanoferrites, which resemble ceramics owing to their chemical formula (AB_2_O_4_). Because of their intriguing and appealing properties, spinel nanoferrites are used across a variety of technical applications and fundamental research. Examples of these applications include wastewater treatment systems, high-performance energy storage devices, anticancer medication administration, high-frequency and switching operational devices, and magnetic hyperthermia applications.^1^ Nonetheless, due to their high saturation magnetization, ferrite materials are used in transducers, microwave equipment, memory chips, deflection yoke rings, and transformers. The size and conditions of the nanoparticles primarily determine their structural, microstructural, and other characteristics. Consequently, the key factors for innovative applications have been recognized as the resistivity, particle size, shape, magnetic, and porous structure. According to some authors,^2^ rare-earth-doped ferrites have a unique place in the market because of their exceptional properties. They also note that these characteristics heavily depend on the particle size. Rehman *et al.*^3^ discussed the magnetization of 34 A m^2^ kg^−1^, which was obtained in size-regulated Mn–Zn ferrites with crystallite sizes ranging between 20 and 80 nm. Significantly, studies have demonstrated that silver nanoparticles exhibit broad-spectrum antibacterial activity, which can eliminate a range of common bacteria, including some that are resistant to antibiotics.^4^ Ag NPs have been suggested for inactivating the bacteria in drinking water to prevent the formation of dangerous DBPs, as they exhibit a gradual, long-lasting, and persistent bactericidal action.^5^ Additionally, it has been demonstrated that the bactericidal effect of Ag NPs is highly size-dependent, increasing with decreasing particle size. Ag particles smaller than 30 nm tend to aggregate, which lowers their antibacterial effectiveness.^6^ Ag NPs can be added to suitable organic or inorganic matrices to reduce aggregation.^7^ This could maintain a high surface-to-mass ratio of Ag NPs, which is essential for their potent antibacterial activity.^8^

Graphene is created when a single, closely packed monolayer of carbon atoms in two dimensions forms a honeycomb network of sp^2^-hybridized carbon atoms.^9^ Recently, graphene has garnered considerable interest in different studies because of its extraordinary qualities, which include high electron mobility, remarkable mechanical stiffness, improved electronic transport, and high electrical conductivity.^10^ Along with the above-mentioned physical and chemical features, graphene has also recently been studied for biological applications; the nanomaterial possesses great biological qualities, like antimicrobial,^11^ biosensing,^12^ cellular imaging, and the ability to administer medications,^13^ in addition to exhibiting anticancer properties.^14^ The ability of graphene or graphene oxide (GO) to prevent cancer has been documented in only a few studies to date. The antitumor properties of a new GO–hypocrellin, compared with free hypocrellin A in an aqueous solution, showed that the hybrid was more effective. GO–TiO_2_ hybrid demonstrated good photo-dynamic anticancer action without dark cytotoxicity; it also significantly increased caspase-3 activity, causing apoptosis.^15^

Ag NPs have been used in retinal endothelial cells as antiangiogenic and anticancer agents according to several studies. Ag NPs exhibited unexpected toxicity in two human cell lines: *human glioblastoma cells (U251)* and *human lung fibroblast cells (IMR-90)*,^16^ as well as endothelial cells^17^ and human breast cancer cells (MDA-MB-231).^17^ However, compared to graphene–silver nanocomposites, Ag and graphene have lower biological activity. Pasricha *et al.*^19^ reported that Ag NPs, halloysite nanotubes, and graphene nanocomposites demonstrated improved antibacterial activity compared to *Staphylococcus aureus* and *Escherichia coli*. Previous studies have reported the toxicity of graphene oxide (GO) to cells and its associated compounds in human cells, such as PC12 cells derived from brain pheochromocytoma,^20^ fibroblasts,^21^ human lung epithelial cells,^21^*MCF-7* cells,^14^ and human breast cancer cells *MDA-MB-231*.^18^ The application of graphene–Ag NP in ovarian cancer cells has not yet been thoroughly studied. Some authors have reported that Ag NP-decorated graphene also exhibits enhanced antibacterial activity.^19,20^ Today, silver is utilized in many dental procedures, catheters, and burn wounds to inhibit the growth of microorganisms. Ag ions exhibit severe biocidal effects on 12 kinds of bacteria, including *Escherichia coli*, demonstrating their toxicity to microorganisms, such as *E. coli*.^21^

Increased medical attention is being paid to the effects of human strains of multiple-resistant bacteria and fungi on commonly prescribed antimicrobial medications, as well as their consequences for public health, increased expenses, and hospitalization. Furthermore, due to their distinct modes of action against cell pathogens, Ag^+^ and Ag nanoparticles display bactericidal and fungicidal effects against resistant strains.^22^ Non-toxic, more potent antimicrobial medicines are desperately needed in pharmacology and toxicology to oppose bacterial infections, particularly those caused by MDR (multiple-drug-resistant) clinical strains.^23–25^ In this regard, Roy *et al.*^26^ revealed that skin exposed to Ag NPs did not change in appearance or structure; these results suggest that green, biosynthesized Ag NPs offer a quick, non-toxic way to create a more potent product. In this study, (Ag_*x*_Zn_1−*x*_Fe_1.95_Ce_0.05_O_4_/10% graphene) NCs (*x* = 0, 0.025, 0.05, 0.075, and 0.1) were created using a sol–gel/auto-combustion technique. To understand the effect of Ag^+^ on the structural, microstructural, and magnetic properties, as well as the antimicrobial, antioxidant, and anticancer activities of the produced novel compositions in nanosized form, experiments with XRD, TEM, and VSM were conducted and discussed in detail.

Significantly, this study offers a thorough structure–property–bioactivity relationship, showing that optimized Ag content (*x* = 0.05–0.1) produces an impressive reduction in IC_50_ (up to 80%) and improved antibacterial efficiency, outperforming the performance of individual components like pure Ag NPs. To the best of our knowledge, this is one of the first reports to combine Ag doping, Ce substitution, and graphene (as a conductive and high-surface area material) to hybridize Zn-ferrite systems for biomedical applications and simultaneous magnetic tuning. This opens up new avenues for creating sophisticated multifunctional nanomaterials for biomedical applications.

## 2 Experimental part

### 2.1. Preparation of Ag_*x*_Zn_1−*x*_Fe_1.95_Ce_0.05_O_4_/10% graphene NCs

**Fig. 1:**
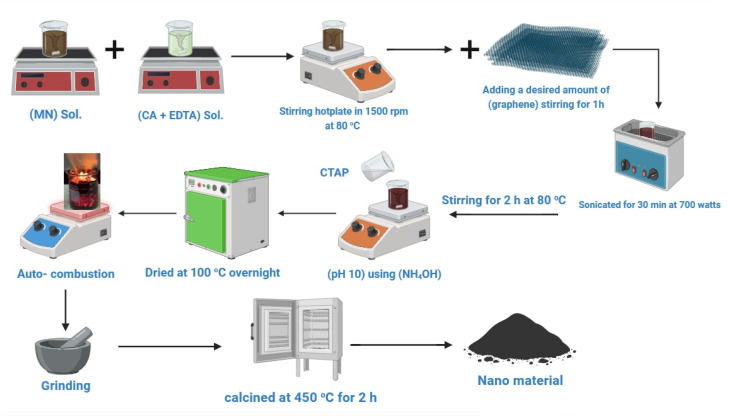
Schematic of preparation route of Ag_*x*_Zn_1−*x*_Fe_1.95_Ce_0.05_O_4_/10% graphene nanostructures using the auto-combustion/sol–gel method.

Ag_*x*_Zn_1−*x*_Fe_1.95_Ce_0.05_O_4_/10% graphene (*x* = 0, 0.025, 0.05, 0.075, and 0.1) ferrite nanostructures are produced using an auto-combustion/sol–gel process. Stoichiometric amounts of Ag(NO_3_), Zn(NO_3_)_2_·6H_2_O, Fe(NO_3_)_3_·9H_2_O, and Ce(NO_3_)_3_ were first dissolved in a minimum amount of double-deionized water to prepare solution I. Citric acid (C_6_H_8_O_7_) and EDTA were dissolved to create solution II in a minimal volume of DW (citric acid : EDTA : metal nitrate) (CA : EDTA : MN) molar ratios were (2 : 1 : 1). Solutions I and II were mixed in another beaker and placed on a stirring hotplate at 1500 rpm and 80 °C until homogenization occurred. After an hour of stirring, a predetermined quantity of graphene was carefully added to the mixture. This combination was vigorously sonicated at 700 watts for 30 minutes and stirred for 2 hours at 80 °C. Then, 100 mL of CTAB was added, and ammonia (NH_4_OH) was used to adjust the pH to 10. The homogenous solution was stirred and heated until it was transformed into a gel and dried at 100 °C overnight. The mixture was placed on a hotplate that had been heated to its highest setting; no auto-ignition or self-propagating combustion was observed. The product was ground for half an hour and calcined at 450 °C for 2 h (Fig. 1).

### 2.2. Instrumental characterization

The crystallinity and structure of Ag_*x*_Zn_1−*x*_Fe_1.95_Ce_0.05_O_4_/10% graphene (*x* = 0, 0.025, 0.05, 0.075, and 0.1) NCs were analyzed using Philips X'pert powder XRD with monochromatic CuK_α_ radiation with a wavelength of 1.54 Å and a step of 0.02°. XRD patterns were submitted for crystal structure analysis using the Highscore Plus software. The surface morphology and nano sizes are investigated and collected using an SEM (JEOL, JSM-6360LA, Tokyo, Japan) instrument operated at 15 kV. The EDX unit, as an accessory to the previous device, was used to determine the elemental composition. HR-TEM and SAED are used (JEOL, JSM-2100 PLUS, Japan) at an accelerating voltage of 200 kV. A vibrating sample magnetometer (7400-1 VSM, U.S., Lakeshore Co., Ltd, USA) with a maximum applied field of ±20 kOe was used to measure the magnetic properties of the samples under ambient conditions.

### 2.3. Antimicrobial, antioxidant and anticancer activities

This study section was conducted at the Regional Center for Mycology and Biotechnology (RCMB), Al-Azhar University, Cairo. The antimicrobial activity of the samples was determined using the agar-diffusion method. The bacterial inoculum concentration was adjusted to the 0.5 McFarland standard (∼1–2 × 10^8^ CFU mL^−1^). A well with a diameter of 6.0 mm was filled with 100 µL of a sample of 10 mg mL^−1^. The efficiency of the sample against fungi was compared with that of the *positive control, ketoconazole*, at a concentration of 100 µg mL^−1^. Similarly, the sample's activity against bacteria was evaluated using a *positive control, gentamicin*, at a concentration of 4 µg mL^−1^. During incubation, inhibition zones were formed around the sample, clearly indicating its antimicrobial activity, and were measured in millimeters. For each condition, the assay was performed in three independent biological replicates.

Using the DPPH free radical scavenging assay, the antioxidant activity of the prepared NCs was assessed. The assay was run in triplicate, and the analysis used average values. To commence the assay, a freshly prepared methanolic solution of 2,2-diphenyl-1-picrylhydrazyl (DPPH) (0.004% w/v) kept in the dark at 10 °C. Additionally, a methanol solution of the test chemical was prepared. To experiment, 3 mL of DPPH solution was mixed with an aliquot of 40 µL of methanol solution containing the test drug. Immediately after mixing, absorbance measurements were monitored using a UV-visible spectrophotometer (Milton Roy, Spectronic 1201). Mixture absorbance was measured at 515 nm at 1 minute intervals for a total of 16 minutes or until the absorbance stabilized. To determine antioxidant activity, the decrease in the absorbance of the DPPH radical in the presence of the tested compound was compared with a control containing only the DPPH radical without any antioxidant. For comparison, the absorbance of ascorbic acid, the reference ingredient, was also measured. Three duplicates of each measurement were made, and the average values were established. The inhibition (PI) % of the test compound of the DPPH radical was estimated using the following equation:PI = [((control absorbance − sample absorbance)/control absorbance) × 100].

This computation gives a measure of the extract's antioxidant activity.^26^

The human hepatocellular carcinoma cell line used to evaluate cytotoxicity against *HepG-2* cells was obtained from the American Type Culture Collection (ATCC, Rockville, MD). Chemicals used are as follows: trypan blue, MTT, fetal bovine serum, and dimethyl sulfoxide (Sigma, St. Louis, Mo., USA). We bought RPMI-1640, l-glutamine, gentamicin, HEPES buffer solution, and 0.25% trypsin–EDTA from Lonza, Belgium. Cell line propagation: cells were cultured on RPMI-1640 media enhanced with 50 µg mL^−1^ of gentamicin and 10% inactivated fetal calf serum. Cells were subcultured 2 or 3 times per week and maintained at 37 °C in a humidified environment with 5% CO_2_. Viability assay for cytotoxicity evaluation: in Corning® 96-well tissue culture plates, tumor cell lines were suspended in medium at (5 × 10^4^) cells per well for antitumor assays, and plates were incubated for 24 h. Then, the examined samples were included in three duplicate 96-well plates, yielding eight concentrations for each sample. For each 96-well plate, six vehicle controls, including media or 0.5% DMSO, were run. An MTT assay was carried out to determine the number of viable cells after 24 hours of incubation.

In summary, the medium from the 96-well plates was removed and replaced with 100 µL of fresh RPMI 1640 culture medium free of phenol red. Each well, with untreated controls, also received 10 µL of 12 mM MTT stock solution, which was set up by dissolving 5 mg of MTT in 1 mL of PBS. The 96-well plates were incubated for 4 h at 37 °C with 5% CO_2_. 50 µL of DMSO was added to each well after an 85 µL aliquot of the media was taken out of the wells. The wells were then well-mixed using a pipette, and the media were incubated at 37 °C for 10 minutes. The number of viable cells was then established by determining the optical density at 590 nm using a microplate reader (SunRise, TECAN, Inc., USA). The experiments were performed in triplicate.Viability (%) = [(*O*_Dt_/*O*_Dc_)] × 100%,where *O*_Dt_ is the average optical density of the wells treated with the tested substance. The mean optical density of the untreated cells is known as *O*_Dc_. The survival curve of each tumor cell line following treatment with the designated substance is achieved by plotting remaining cells *vs.* drug concentration. Using the Origin software, the 50% inhibitory concentration (IC_50_) needed to produce harmful effects in 50% of intact cells was calculated from the dose–response curve visual plots for each concentration.

## 3 Results and discussion

### 3.1. XRD analysis

**Fig. 2:**
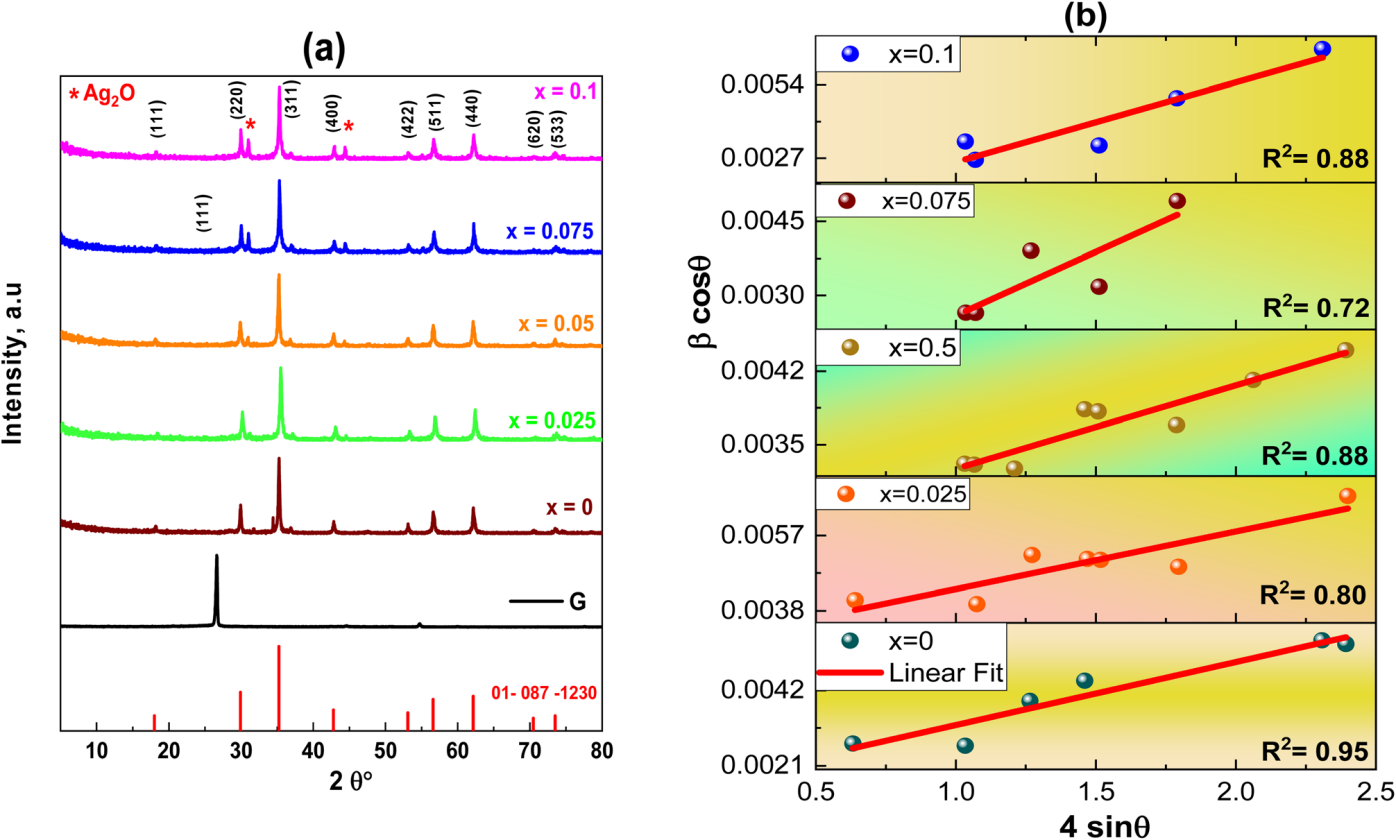
(a) XRD patterns of graphene and the Ag_*x*_Zn_1−*x*_Fe_1.95_Ce_0.05_O_4_ nanoparticles (*x* = 0, 0.025, 0.05, 0.075, and 0.1). (b) Williamson–Hall plots for the Ag_*x*_Zn_1−*x*_Fe_1.95_Ce_0.05_O_4_/10% graphene NCs (*x* = 0, 0.025, 0.05, 0.075, and 0.1), showing the linear fit of data and *R*^2^ values for each fit.

Fig. 2a shows XRD patterns, where Ag_*x*_Zn_1−*x*_Fe_1.95_Ce_0.05_O_4_/10% G (*x* = 0, 0.025, 0.05, 0.075, and 0.1) ferrites present the crystallographic indices (111), (220), (311), (400), (4 2 2), (5 1 1), (4 4 0), (620) and (5 3 3) with diffraction angles between 5° and 80°, indicating a cubic spinel crystalline structure with an *Fd*3̄*m* space group. A small number of diffraction peaks are also observed, which is consistent with earlier reported findings and suggests the presence of silver oxide (Ag_2_O).^27^Eqn (1) was then used to determine the lattice parameters of each sample, where the interplanar spacing, *d*_*hkl*_, was derived using *nλ* = 2*d* sin *θ* as follows:1
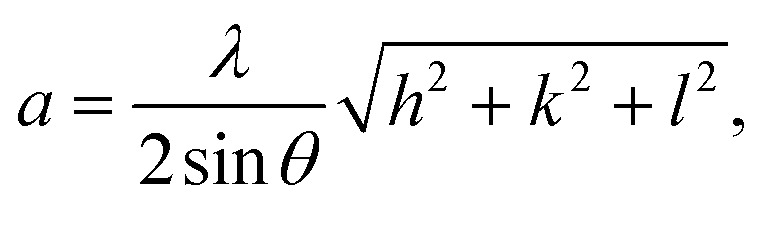
where *λ* = 1.54056 Å is the X-ray wavelength and a is the lattice parameter in Å. The peak's interplanar spacing with respect to the crystallographic plane under study is denoted by the symbol *d*_*hkl*_, *θ* is the diffraction angle, and *hkl* are Miller indices corresponding to 2*θ*. The average intensity of all Bragg reflections was obtained to determine the mean crystallite size using Scherrer's equation (eqn (2)). The results are presented in Table 1, where *β* represents full width at half maxima and *θ* is Bragg's angle as follows:2
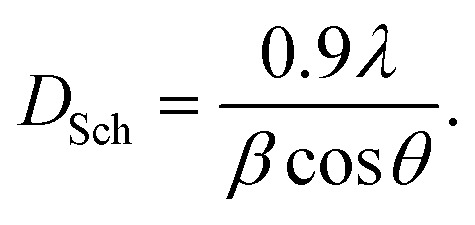


**Table 1:** Structural parameters of Ag_*x*_Zn_1−*x*_Fe_1.95_Ce_0.05_O_4_/10% G (*x* = 0, 0.025, 0.05, 0.075, and 0.1)

Samples	*a* (Å)	*V* (Å)^3^	CS (nm)	*D*_(W–H)_ (nm)	Grain size (nm)	*ε* × 10^−3^	*δ* × 10^−4^ (nm^−2^)
*x* = 0.00	8.4375	600.67	49.87	99	57.00	1.7	4.02
*x* = 0.025	8.3882	590.21	41.38	49.5	54.50	1.4	5.84
*x* = 0.05	8.4421	601.66	35.81	57.7	46.45	0.7	7.80
*x* = 0.075	8.4221	597.40	45.11	92.4	57.60	2.5	4.91
*x* = 0.100	8.4273	598.50	41.05	36.48	59.70	2.9	5.93

Together with microstructural strain, another method employed to assess crystallite size is the Williamson–Hall equation (eqn (3)) (Fig. 2b) as follows:3
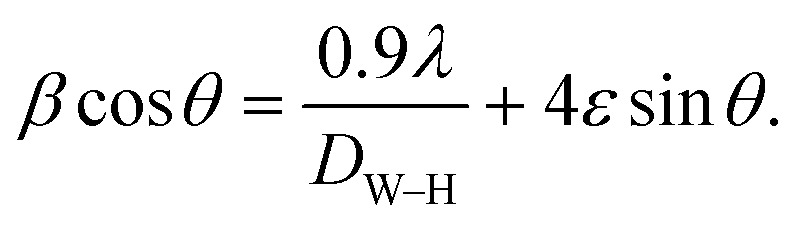


**Fig. 3:**
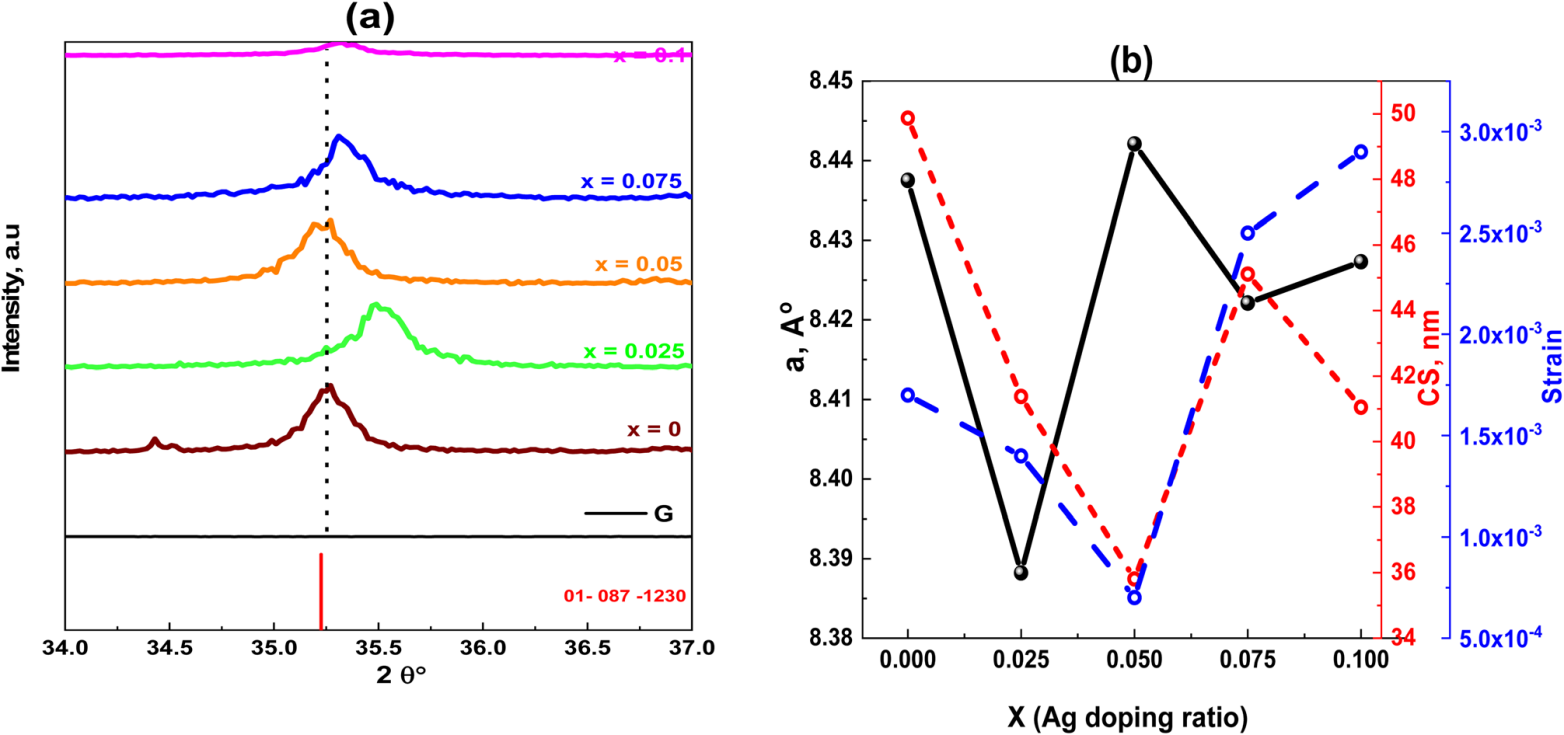
(a) Effect of Ag doping ratio on the shift of the most prominent peak position. (b) Ag dependence on the lattice parameters, crystallite size and strain of Ag_*x*_Zn_1−*x*_Fe_1.95_Ce_0.05_O_4_/10% G NCs (*x* = 0, 0.025, 0.05, 0.075 and 0.1).

The determined size of the crystallites (*D*) for every synthesized sample is recorded in Table 1, which makes it evident that the crystallite size (*D*) varies with Ag^+^ doping, ranging from 36.48–99 nm. Ce^3+^ cations have larger cationic radii (1.143 Å) than Fe^3+^ cations (0.645 Å), and the activation energy needed to reside in the octahedral state is higher than that of Fe^3+^ cations,^28^ where the cause might be a stronger Ce–O bond energy than the Fe–O bond energy. The existence of graphene and variations in the behavior of various lattice parameters, which appear in the shifting of 2*θ* (Fig. 3a), are caused by incorporating dopant Ag ions in the spinel matrix and variation in cationic radii, which exhibit a linear dependency and obey Vegard's rule.^29^Fig. 3b shows the crystallite size, lattice constant, and strain *versus* the Ag content.

The formation of *ε* within the lattice is attributed to the ionic size disparity between Ag^+^ and Zn^2+^ in comparison to Ce^3+^ and Fe^3+^, as illustrated in Table 1. As a consequence, localized structural disorder ensues, slowing nucleation rates due to reduced crystallite dimensions. The dislocation density (*δ*) is measured to evaluate the quality of the samples. Eqn (4) is used to determine the dislocation or quantity of dislocated lines per unit volume of the crystal lattice. The following equation is used to study dislocation line density (*δ*):4
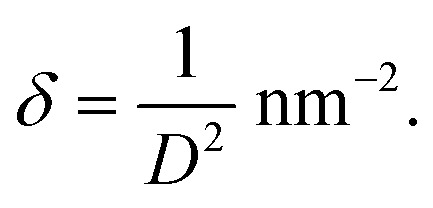


Table 1 indicates that samples with smaller crystallite sizes exhibit a greater increase in *δ*. This finding is consistent with the finding of ref. 32, which states that as *δ* increases, crystallinity declines and crystal granules become smaller.

It is possible to estimate the bond angle, interionic distance, and oxygen positional parameter. Because of the different ionic radii of cations, the tetrahedral site expands more than the octahedral site when divalent ions are present in tetrahedral interstices. The quantitative measure of oxygen-ion displacement, provided by the oxygen positional parameter *u*, can explain this growth. Anytime the radii of the replaced and substituted ions diverge, this displacement occurs. Using eqn (4)–(6), one can determine the oxygen positional parameter *u* based on the theoretical lattice parameter, *a*_th_, as follows:5
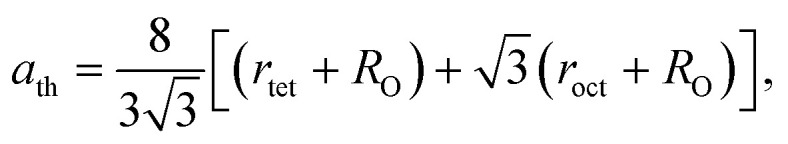
where the oxygen ionic radius (*R*_O_) = 1.32 Å.6
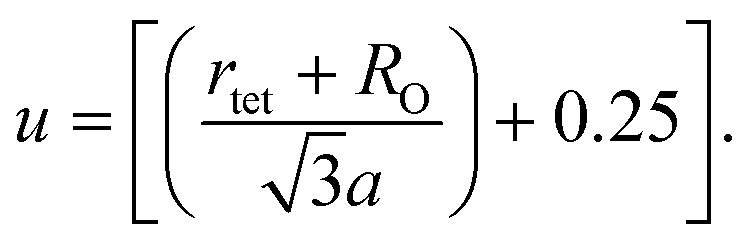


The optimal value of *u* in a cubic spinel structure is 3/8 = 0.375. Anion's displacement from its ideal position might be the reason the computed value of *u* is marginally greater than the ideal value. It is verified by analyzing *u* and *δ* (=*u* − 0.375) that the lattice is somewhat deformed for every sample in both series.^30^ The hopping length (*L*_A_ and *L*_B_) values obtained from Stanley's equations (eqn (7) and (8))^30^ are presented in Table 2 as follows:7
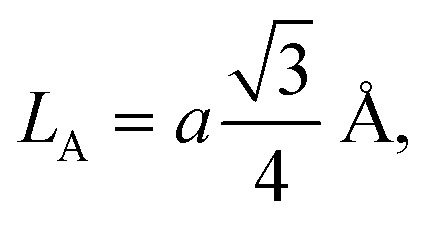
8
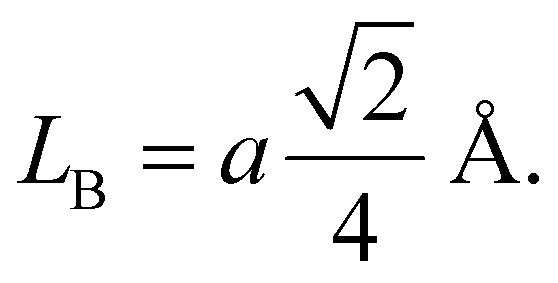


**Table 2:** Hopping lengths (*L*_A_ and *L*_B_), tetrahedral bond length (*d*_LA_), octahedral bond length (*d*_LB_), interstitial radii (*r*_A_ and *r*_B_), and tolerance factors of Ag_*x*_Zn_1−*x*_Fe_1.95_Ce_0.05_O_4_/10% G

*x*	*A*, Å	*L*_A_, Å	*L*_B_, Å	*d*_LA_, Å	*d*_LB_, Å	*r*_A_, Å	*r*_B_, Å	*T*
0	8.4375	3.653	2.983	1.89	2.06	0.57	0.74	1.02
0.025	8.3882	3.632	2.965	1.94	2.05	0.62	0.73	1.03
0.050	8.4421	3.655	2.984	1.95	2.06	0.63	0.74	1.03
0.075	8.4221	3.646	2.977	1.89	2.06	0.57	0.74	1.02
0.1	8.4273	3.647	2.979	1.89	2.06	0.57	1.02	1.02

The following formulas were utilized to compute the respective bond lengths of the tetrahedral and octahedral interstitial lattice positions (eqn (9) and (10)), and the results are presented in Table 2 as follows:9
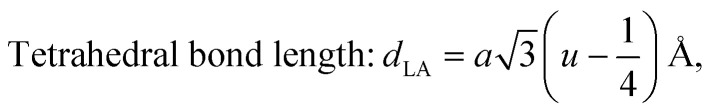
10
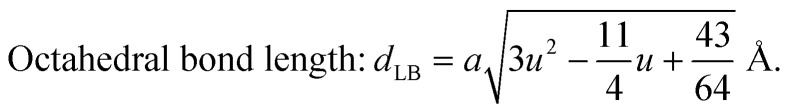


Eqn (11) and (12) were utilized to calculate the ionic radii (*r*_A_ and *r*_B_) of the tetrahedral and octahedral interstitial lattice sites, which are presented in Table 2 as follows:11
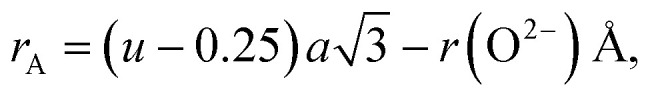

12 *r*_B_ = (0.625 − *u*)*a* − *r*(O^2−^) Å.

Eqn (13) is used to calculate the tolerance factor^31^ as follows:13
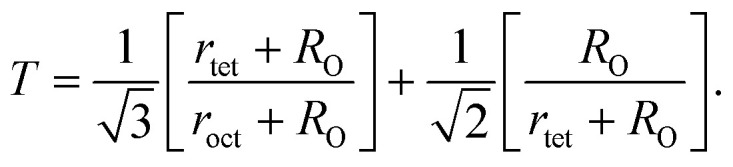


### 3.2. SEM investigation

**Fig. 4:**
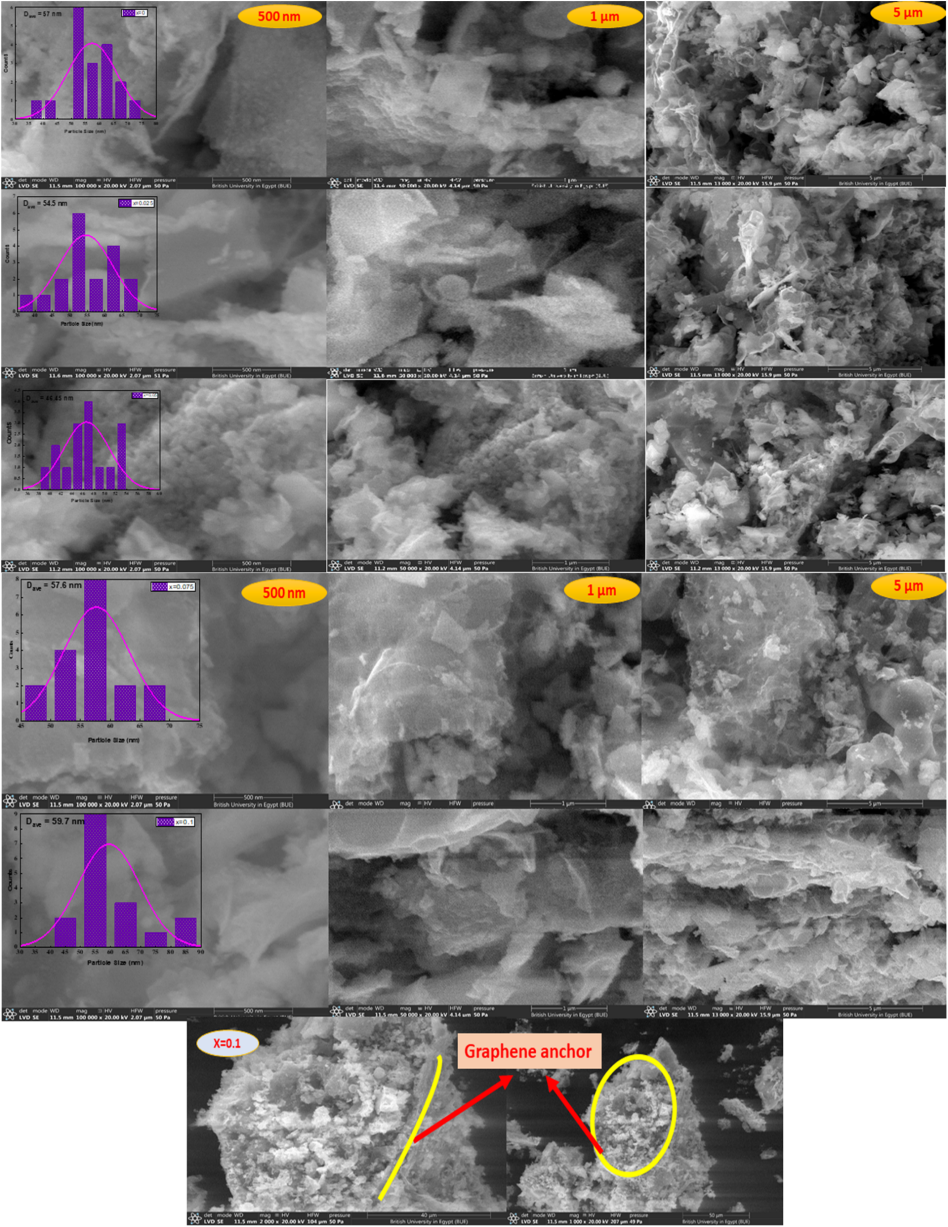
SEM micrographs and histogram of Ag_*x*_Zn_1−*x*_Fe_1.95_Ce_0.05_O_4_/10% G (*x* = 0, 0.025, 0.05, 0.075, and 0.1) ferrites under various magnifications (500 nm, 1 µm, and 5 µm). The bottom images are for the sample *x* = 0.1 at 40 and 50 µm.

SEM micrographs of Ag_*x*_Zn_1−*x*_Fe_1.95_Ce_0.05_O_4_ (*x* = 0, 0.025, 0.05, 0.075, and 0.1) nanoparticles, along with their histograms, are depicted in Fig. 4. The nanoparticles had an average size of about 46.45–59.70 nm. The grain size of five samples at three magnifications (500 nm, 1 µm, and 5 µm) is represented. The estimated XRD crystallite sizes and the projected SEM particle sizes correlate rather well. Particle sizes vary irregularly with increasing Ag^+^-ion concentration. The images reveal nanoparticles with homogeneous spherical particles anchored to graphene nanosheets.

### 3.3. HR-TEM and SAED analyses

**Fig. 5:**
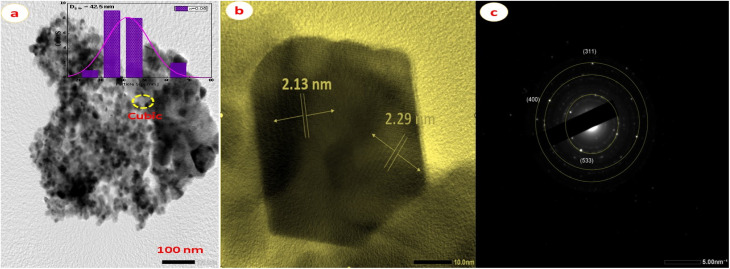
(a) Typical TEM image, (b) HR-TEM image, and (c) SAED pattern of the (*x* = 0.05) sample of Ag_0.05_Zn_0.95_Fe_1.95_Ce_0.05_O_4_/10% G NCs.

The TEM image is shown in Fig. 5a. The particle size is found to be 42.5 nm of Ag_0.05_Zn_0.95_Fe_1.95_Ce_0.05_O_4_/10% G, which are nearly spherical nanocrystallites, confirming the SEM images. There is little aggregation and an uneven form in the images. Comparing the diameters determined by XRD and TEM reveals a negligible distinction. This discrepancy in crystallite size between XRD and TEM evaluations is attributed to XRD providing a crystallite size. In contrast, TEM assesses particle size, which may contain a composite of multiple crystallites. The HR-TEM images of Ag_0.05_Zn_0.95_Ce_0.05_Fe_1.95_O_4_/10% G include well-defined two-dimensional lattice spaces in polycrystalline planes that vary in crystal direction by 2.13 nm and 2.29 nm for spinel ferrites in lattice planes (400) and (3 1 1), respectively, as shown in Fig. 5b. Moreover, the SAED pattern (Fig. 5c) on an individual *x* = 0.05 sample represents the lattice fringes of Ag_0.05_Zn_0.95_Fe_1.95_Ce_0.05_O_4_/10% G belonging to the crystalline planes of the cubic structure, which is steady with the obtained XRD pattern. The high degree of crystallinity of the samples is indicated by the presence of distinct dot circles in the SAED pattern.^34^ The initial three SAED pattern rings were designated as follows: (3 1 1), (4 0 0), and (5 3 3). The data from selected-area diffraction, as shown in Fig. 5c, correspond to the lattice planes of the *Fd*3̄*m* cubic phase and are therefore identical to the XRD results. The reflection at (3 1 1) had the highest intensity, corresponding to the XRD pattern shown in Fig. 2a.

### 3.4. EDAX analysis and mapping images for *x* = 0.1

**Fig. 6:**
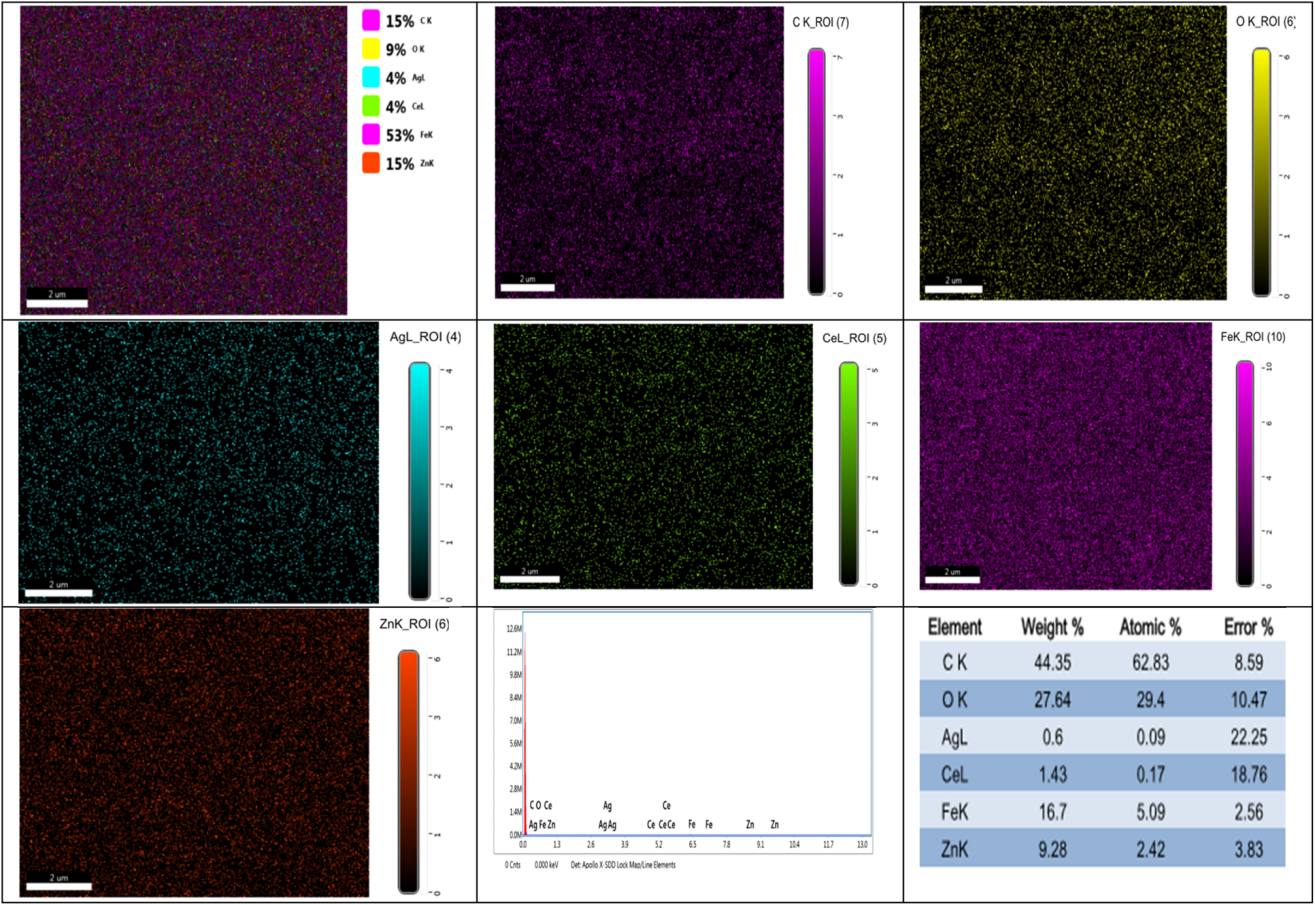
EDX spectrum and elemental mapping images of Ag_*x*_Zn_1−*x*_Fe_1.95_Ce_0.05_O_4_/10% G NCs (*x* = 0, 0.025, 0.05, 0.075, and 0.1).

The homogeneity, stoichiometry, and chemical makeup of the materials generated were investigated using EDAX analysis, along with the corresponding elemental mapping images, as illustrated in Fig. 6. No impurities were identified in the EDAX spectrum, which confirmed the integrity of the synthesized samples by revealing prominent Ag, Zn, Ce, Fe, and O peaks. Furthermore, the elements are distributed uniformly, as evidenced by the mapping images, suggesting that the samples have been adequately prepared. Data from EDX verified the creation of pure nanostructured Ag_*x*_Zn_1−*x*_Fe_1.95_Ce_0.05_O_4_/10% graphene (*x* = 0.05) samples. These findings support the successful fabrication of the spinel cubic phase structure, as observed in the XRD and FTIR findings.

### 3.5. FT-IR analysis

**Fig. 7:**
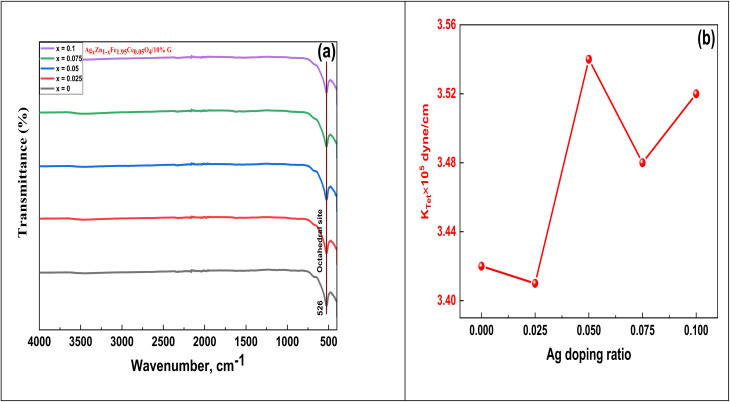
(a) FT-IR spectra and (b) force constant as a function of Ag doping ratio in the Ag_*x*_Zn_1−*x*_Fe_1.95_Ce_0.05_O_4_/10% G NCs.

FTIR spectra of the prepared samples of Ag_*x*_Zn_1−*x*_Fe_1.95_Ce_0.05_O_4_/10% G in the range of *ν* 4000–400 cm^−1^ are shown in Fig. 7a. These spectra are used to determine whether other functional groups are present in ferrite and how spinel structures arise. Based on the arrangement of oxygen atoms in the FCC structure, ferrites have metal ions occupying two distinct interstitial positions in the lattice: tetrahedral site (A-site) and octahedral site (B-site). As per reports, the A-site is associated with a band observed in the high-frequency range of *ν* 600–500 cm^−1^. Conversely, the octahedral B-site is linked to the band observed in the low-frequency range of *ν* 450–385 cm^−1^.^32^ Stretching vibrations of the A-site tetrahedral metal–oxygen bond are assigned to the high-frequency band at about *ν* 526 cm^−1^. With increasing Ag-content, this band shifted sluggishly toward higher frequencies in the remaining samples. However, all the samples exhibited any signal in the lower frequency band, typically detected within the range of *ν* 450–385 cm^−1^, which is ascribed to vibrations of octahedral metal–oxygen.^33^ These increments and shifts in the *ν* values are also clearly illustrated in Table 3. The bands at approximately *ν* 3470 and 1600 cm^−1^, which indicate stretching and bending modes of H–O–H, respectively, are not present, giving the impression of the absence of hydration or absorbed water in the samples. Additionally, the band at *ν* 1400 cm^−1^ is a result of the antisymmetric NO stretching vibrations, which are caused by the residual nitrate group in samples. These bands are absent, indicating that the samples contain an extremely tiny quantity of impurity.

**Table 3:** Values of the force constant *k*_T_ and infrared vibrational mode frequency (*ν*_tetra_) for the Ag_*x*_Zn_1−*x*_Fe_1.95_Ce_0.05_O_4_/10% G NCs (*x* = 0, 0.025, 0.05, 0.075, and 0.1)

*x*	*ν*_tetra_ (cm^−1^)	*k*_T_ × 10^5^ (Dyne per cm)
0.000	526	3.42
0.025	527	3.41
0.050	528	3.54
0.075	529	3.48
0.100	530	3.52

By entering values of *ν* in the following relations, force constant parameters related to the interstitial tetrahedral sub-lattice (*k*_tetra_) were computed as follows:^34^

14 *k*_T_ = 4π^2^*c*^2^*ν*^2^*µ*.

In this equation, 
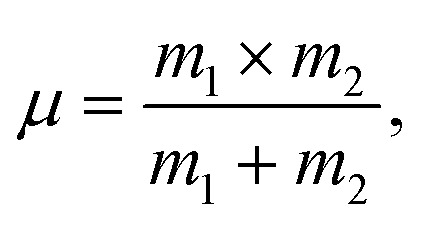
 where *m*_2_ is the atomic weight of the oxygen anion and *m*_1_ is the average weighted of cation atomic weights at the A- or B-site. Table 3 includes the compositional variations for these values. Table 3 clearly illustrates that, with the substitution of Ag^+^-ions, all force constant values decrease in a compositional manner, as shown in Fig. 7b. This trend was brought on by the location of cationic redistribution in the zinc ferrite spinel matrix after the Ag^+^ ion substitution. Ag^+^ ions choose the octahedral [B] sub-lattice for accommodation. According to Dhanda *et al.*, this kind of alteration is caused by Fe^3+^ ions moving from the interstitial octahedral [B] sub-lattice to the tetrahedral (A) sub-lattice.^35^ The outcome is an imbalance at the [B] sub-lattices that encourages O^2−^ ions to shift towards Fe^3+^ ions, leading to a decrease in the force constant parameters. At around *ν* 3000 cm^−1^, an ambiguous absorption band is observed (*v*_3_). This could be linked to H–O–H stretching vibrations, as moisture did not affect the investigated samples during production.^36^

### 3.6. Magnetic properties study

**Fig. 8:**
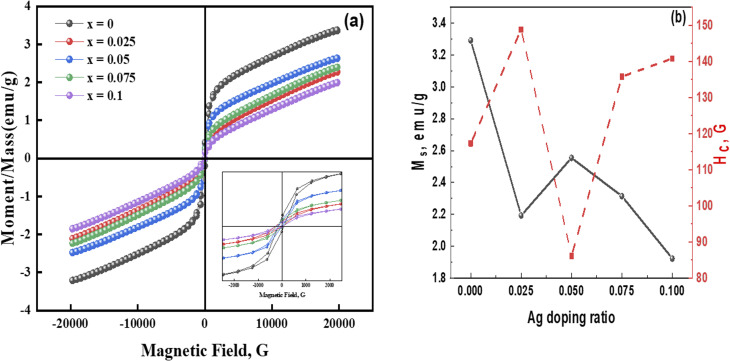
(a) VSM measurement of Ag_*x*_Zn_1−*x*_Fe_1.95_Ce_0.05_O_4_/10% G NCs (*x* = 0, 0.025, 0.05, 0.075, and 0.1) at room temperature and magnetic field of ±20 kOe. (b) Ag doping ratio dependence on the magnetization and coercivity.

A room temperature VSM approach was used to obtain magnetization (*M*) *versus* the applied magnetic field (*H*) range of ±20 kOe plots, as illustrated in Fig. 8a. Table 4 provides the magnetic characteristics determined from the plots, including the squareness ratio (SQR = *M*_r_/*M*_s_), coercive field (*H*_c_), remanence magnetization (*M*_r_), and saturation magnetization (*M*_s_) for each sample. The magnetization of these samples did not reach saturation even at 20 kOe of the applied magnetic field. Given the shape of the curves, it might be inferred that the samples exhibit a mixture of ferrimagnetic and paramagnetic properties. Sample *x* = 0 (no Ag) shows a saturation magnetization of 3.29 emu g^−1^ and a coercive field of 117.3 Oe. When Ag is added, sample *x* = 0.025 exhibits a similar hysteresis loop, with a coercive field increase to 148.80 Oe and a reduction in *M*_s_ to 2.19 emu g^−1^. However, when Ag-content is doubled, the coercive field's value drops sharply to 56%. The coercive field rises to 140.83 Oe of sample *x* = 0.1, while the saturation magnetization falls to 1.92 emu g^−1^. As the amount of Ag increases, the paramagnetic contribution decreases, and the magnetic parameters exhibit unsystematic behavior due to the presence of graphene in the samples. *H*_c_ was found to be independent of the particle size in the current samples, which could be connected to graphene's existence. The anticipated shift from single-domain to multi-domain action may be the cause of the decrease in coercivity. This behavior can be explained by Stoner–Wohlfarth's (S–W) single-domain hypothesis.^37^ However, as particle size increases in the multi-domain zone, coercivity tends to decrease. This discrepancy is ascribed to the presence of graphene. A value of SQR less than 0.5, according to S–W theory, indicates the creation of multi-domain structures. This outcome suggests that samples with SQR values below 0.5 might be due to surface spin disorder. Fig. 8b illustrates how magnetization and coercivity depend on Ag-content.

**Table 4:** Magnetic parameters of the Ag_*x*_Zn_1−*x*_Fe_1.95_Ce_0.05_O_4_/10% G NCs (*x* = 0, 0.025, 0.05, 0.075, and 0.1)

*x*	*M*_s_, emu g^−1^	*H*_c_, Oe	*M*_r_, emu g^−1^	*M*_r_/*M*_s_ × 10^−3^	*K*, erg cm^−3^	*n*_B_
0.000	3.29	117.30	0.266	79.92	402.23	0.14
0.025	2.19	148.80	0.105	48.01	339.88	0.09
0.050	2.55	86.124	0.126	49.21	229.19	0.11
0.075	2.31	135.79	0.132	57.10	327.29	0.10
0.100	1.92	140.83	0.073	38.14	270.76	0.08

Eqn (15) was applied to determine the magnetic moment as follows:^35^15
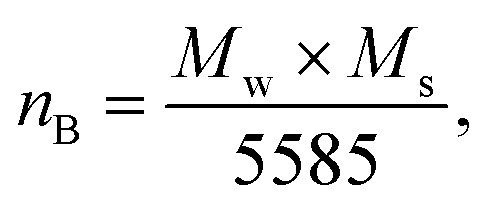
where *M*_w_ and *M*_s_ are the molecular weight and magnetization of the samples, respectively. The magnetic moment (*n*_B_) values decrease as the concentration of Ag increases, which is consistent with the literature.^39^ Using eqn (16), magneto-crystalline anisotropy is determined as follows:^38^16
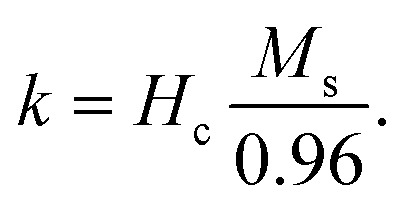


### 3.7. Antimicrobial activity test

Table 5 shows the effect of tested micro-organisms, such as fungi, *Gram-positive* and *Gram-negative* bacteria, on Ag_*x*_Zn_1−*x*_Fe_1.95_Ce_0.05_O_4_/10% G (*x* = 0, 0.05, and 0.1) nanocomposite, assessed in terms of inhibition zone. Tested NCs show no activity against fungi, including *Candida albicans* RCMB 005003 (1) ATCC 10231 and *Aspergillus* fumigatus (RCMB 002008). Additionally, NCs are tested against two bacterial pathogens: *Gram-negative* bacteria, such as *Proteus vulgaris* RCMB 004 (1) ATCC 13315 and *Escherichia coli* ATCC 25922, and *Gram-positive* bacteria, such as *Bacillus subtilis* and *Staphylococcus aureus* RCMB 015 (1) NRRL B-543. Our samples show no activity *vs. Staphylococcus aureus* at 0 concentration; however, upon increasing silver ion concentration, the inhibition zone increased. In *Bacillus subtilis*, the effect was clear even at zero concentration due to the antimicrobial properties of ZnO NPs, which damage the cell wall and disrupt DNA replication. Moreover, upon a 32% increase in particle size, the activity of the compound decreased by 12% due to the low penetration power of the particles in the tested compound, which might have induced cell disruption and the death of the bacterial species. The same scenario was observed for *Gram-negative* bacteria: the compound showed no activity against *Proteus vulgaris* at zero concentration, and upon increasing the silver concentration, the inhibition zone increased to 17 mm and then decreased by 12%. Although *Gram-negative* bacteria have a cell wall structure that is more complex than that of *Gram-positive* bacteria, this structure can make them more resistant to certain antimicrobial agents. Silver and zinc compounds have been used for antimicrobial activity because they are believed to interfere with bacterial cell membranes and inhibit cellular functions. Furthermore, the results presented in Table 2 revealed that Ag NP samples are quite effective against two types of bacteria: having a zone of inhibition range of 14–17 mm at the sample concentration of 10 mg mL^−1^ compared to that of Co_0.8_Ag_0.2_Fe_2_O_4_ (ref. 39) having a zone of inhibition (2, 1, 4, and 4 mm) at the sample concentration (25, 50, 75 and 10 mg mL^−1^) of *Escherichia coli*, *Bacillus subtilis*, and *Staphylococcus aureus*, respectively. However, because of their small size and special qualities, nanoparticles have the potential to kill bacteria through a variety of mechanisms, piercing, breaching, and passing through porin proteins in the cell membrane, reducing the efficiency of mitochondria, leading to ribosome disintegration, denaturing vital proteins, causing DNA damage, and generating ROSs (reactive oxygen species), which include O˙, OH, and others. This leads to the breakdown of essential cellular processes, such as transcription, translation, and replication. This also disrupted cell metabolism and caused DNA damage.^35,40^ Jebapriya *et al.*^41^ reported that Ag^+^ and Fe^2+^ can penetrate the outer membrane and develop inside the membrane, where their adherence causes the cell to become unstable and damaged, acting at the level of the cell membrane. When this happens, the cell dies because the membrane becomes more permeable, allowing cellular contents to escape. Research suggests that Ag^+^/Fe^2+^ can interact with sulfur-containing proteins in bacterial cell walls, potentially causing structural damage, such as cell wall rupture. They also noted that the nanoparticle release of silver and ferrous ions might modify metabolic pathways, membranes, and even genetic material through interactions with biological components, which are driven by their size and charge. This gives the impression that the prepared nanoparticles have better antibacterial properties.

**Table 5:** Antimicrobial activity test of the Ag_*x*_Zn_1−*x*_Fe_1._9_5_Ce_0.05_O_4_/10% G NCs (*x* = 0, 0.05, and 0.1)

Tested microorganisms	*x* = 0	*x* = 0.05	*x* = 0.1	Control
	Formed inhibition zone (mm)			
**Fungi**				**Ketoconazole**
*Aspergillus fumigatus* RCMB 002008	NA	NA	NA	17 ± 0.87
*Candida albicans* RCMB 005003 (1) ATCC 10231	NA	NA	NA	20 ± 1.29

**Gram positive bacteria**				**Gentamicin**
*Staphylococcus aureus* ATCC 25923	NA	16 ± 0.89	17 ± 1.21	24 ± 1.15
*Bacillus subtilis* RCMB 015 (1) NRRL B-543	10 ± 0.64	15 ± 1.28	14 ± 0.96	26 ± 1.36

**Gram-negative bacteria**				**Gentamicin**
*Escherichia coli* ATCC 25922	11 ± 0.72	17 ± 1.12	15 ± 0.73	30 ± 1.97
*Proteus vulgaris* RCMB 004 (1) ATCC 13315	NA	16 ± 0.91	15 ± 0.85	25 ± 1.54

### 3.8. DPPH radical scavenging activity

**Fig. 9:**
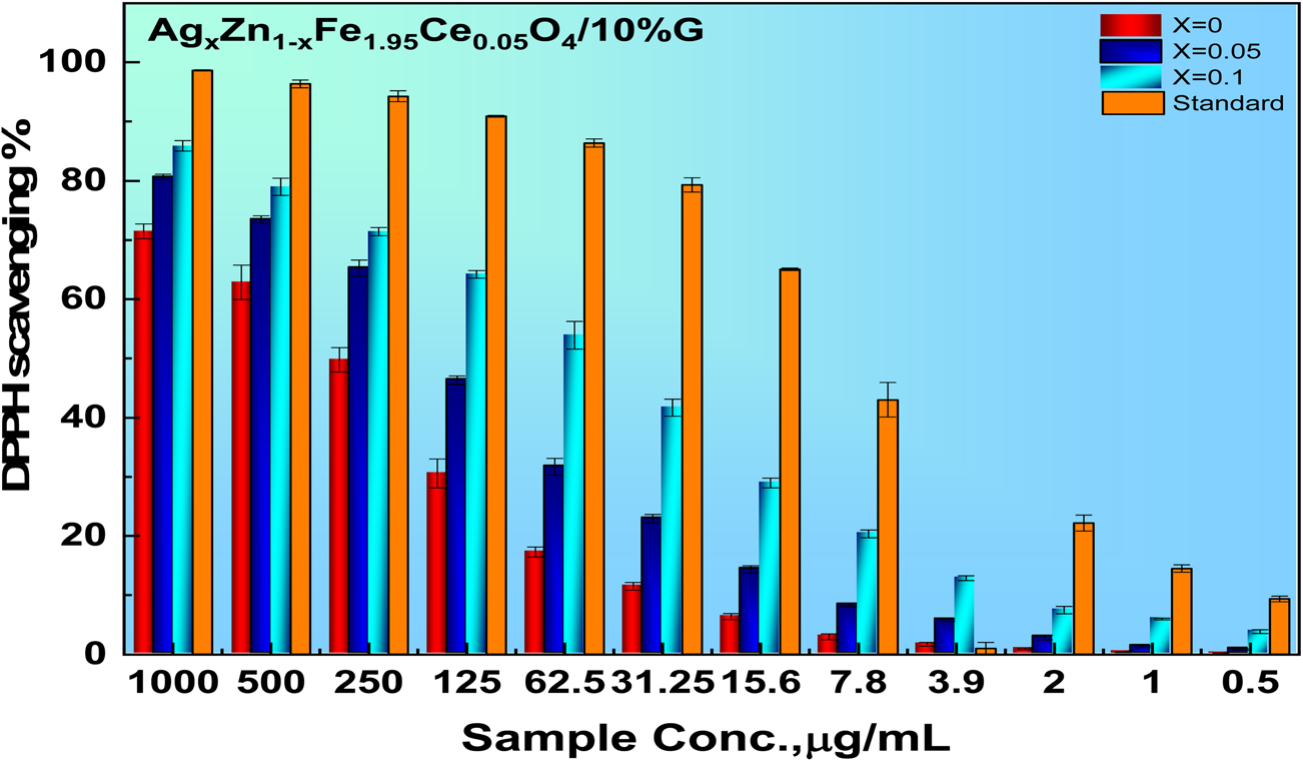
DPPH scavenging dependence on the sample concentration of the Ag_*x*_Zn_1−*x*_Fe_1.95_Ce_0.05_O_4_/10% G NCs (*x* = 0, 0.05, and 0.1).

As depicted in Fig. 9, it is observed that DPPH scavenging activity increased 6% with growing silver ions up to (Ag = 0.1), leading to the highest DPPH scavenging activity as a result of the scavenging effect of silver on free radicals and ROS created during oxidative stress. In addition, zinc ions are essential micronutrients that play a role in defense systems. It is a cofactor for various antioxidant enzymes and helps maintain antioxidant balance by regulating the expression and activity of antioxidant-related genes. In principle, antioxidants neutralize free radicals and prevent oxidation. The generated silver particles demonstrated enhanced radical scavenging activity at varying DPPH concentrations, as illustrated below. Consistent with the findings of some studies,^42,43^ the analysis also suggests that the particle capacity to scavenge increases with increasing molar concentrations of the generated nanoparticles.17
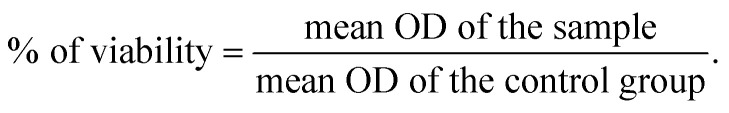


### 3.9. Cytotoxicity test

**Fig. 10:**
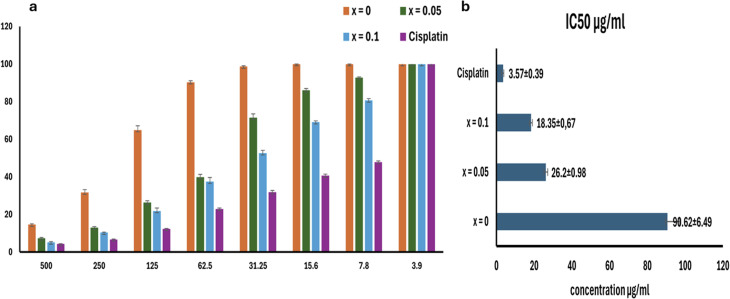
(a) Viability% *vs.* sample concentration of Ag_*x*_Zn_1−*x*_Fe_1.95_Ce_0.05_O_4_/10% G NCs (*x* = 0, 0.05, and 0.1). (b) IC_50_ dependence on the Ag doping ratio. MTT assays were performed in three independent biological replicates, each with technical triplicates; the data are presented as mean ± SEM.

As depicted in Fig. 10a and b, the cytotoxicity test was achieved by MTT assay on the *HepG-2* cell line to examine the effect of silver ions and the nanoparticle compound concentration on cell viability. In this assay, the number of living, not dead, cells existing through MTT-assay exposure is precisely proportional to the amount of formazan produced. As illustrated in Fig. 10a, the concentration of the sample had a remarkable effect on the percentage of cell viability, which increased cell inhibition (94%, 97%, up to 98%). Fig. 10b reveals that the tested nanocompound has a cytotoxic effect on the cell line owing to the presence of silver ions, which affect cell growth and proliferation, leading to cell cycle arrest. The results show that viability decreased by 70% with increasing silver concentration up to *x* = 0.1, with an IC_50_ of 18.35, compared to the control (cisplatin), which has an IC_50_ of 3.57. Silver nanoparticles enter cells, induce apoptosis, and kill cancer cells, and this effect is enhanced by the generation of ROSs. Additionally, there was damage to the cell nucleus and increased cell death. The control cells displayed a full structure and good growth.^44^

Significant biological outcomes are attributed to certain magnetic and structural factors. In terms of structure, the addition of Ag and Ce ions to the spinel ferrite lattice results in defect states, oxygen vacancies, and lattice distortion, all of which are essential for increasing the surface area. Conversely, graphene enhances electron mobility and inhibits nanoparticle aggregation, both of which facilitate the process of charge transfer. These factors encourage the production of reactive oxygen species ROS such as ˙OH and O_2_^−^. Moreover, magnetic domains improve redox cycling at the surface of nanoparticles, increasing the efficiency of ROS formation, which breaks bacterial cell membranes and kills cancer cells.

## 4 Conclusions

Ag_*x*_Zn_1−*x*_Fe_1.95_Ce_0.05_O_4_/10% G (where *x* = 0, 0.025, 0.05, 0.075, and 0.1) nanostructured materials were effectively fabricated using the sol–gel/auto-combustion method. X-ray diffraction patterns identified the cubic phase. Primarily, because of the difference in ionic radii between A and B cations, the distinct crystal structures of the samples vary from 35.81 to 49.87 nm. With a greater magnitude of this disparity, substantial deformation and structures with reduced symmetry are attained. Structural characteristics of samples, such as morphology, agglomeration, and porosity, are likewise discernible through scanning electron microscopy visualizations. Grain size by SEM is 46.45–59.7 nm. The variety of characteristic FT-IR bands validates the crystal structure of the fabricated ferrites. The compound composition, stoichiometry, and homogeneity of the prepared substances were investigated using EDAX analyses and associated elemental mapping images. The lattice parameter estimation for systems accounts for structural types that are members of the space group *Fd*3̄*m*. In addition, the morphology of the nanoparticles observed in the TEM image is 42.5 nm. The HR-TEM images are consistent with the distinct structures observed in the XRD analyses. The high degree of crystallinity of the samples is indicated by the presence of distinct dot circles in the SAED pattern. Additionally, the *M*–*H* hysteresis loops of the examined materials indicated modest ferrimagnetic behavior. *H*_c_ values range from 148.8 to 86.1 Oe. The magnetization *M*_s_ ranges from 3.29 to 1.92 emu g^−1^. The impact of a compound's particle size on its penetration power is substantial, as evidenced by antimicrobial test results, showing 71% reduction in penetration power for both *Gram-positive* strains with the standard gentamicin and 50% reduction for the same standard on *Gram-negative* strains at higher concentrations. Increasing silver concentration synergistically enhanced zinc's cytotoxicity and DPPH scavenging activity in the utilized cell line. There is a 41.5% and 79.5% increase in antioxidant activity and an 80% increase in cytotoxicity.

## Ethical approval

This article does not contain any studies with animals performed by any of the authors.

## Author contributions

Amina Toumi: methodology, investigation, formal analysis, and data curation. Mohammad Abohassan: methodology, investigation, funding acquisition, and formal analysis. H. H. Hegazy: software, investigation, funding acquisition, and formal analysis. Heba Y. Zahran: writing – original draft, visualization, and validation. Elbadawy A. Kamoun: writing – review and editing, writing – original draft, visualization, validation, supervision, and project administration. V. Ganesh: software, resources, methodology, and investigation. Ibrahim S. Yahia: writing – review and editing, writing – original draft, supervision, software, resources, and project administration.

## Conflicts of interest

The authors declare that they have no known competing financial interests or personal relationships that could have appeared to influence the work reported in this paper.

## Data Availability

The datasets used and/or analyzed during the current study are available from the corresponding authors upon reasonable request.
